# Lead Poisoning Disturbs Oligodendrocytes Differentiation Involved in Decreased Expression of NCX3 Inducing Intracellular Calcium Overload

**DOI:** 10.3390/ijms160819096

**Published:** 2015-08-13

**Authors:** Teng Ma, Xiyan Wu, Qiyan Cai, Yun Wang, Lan Xiao, Yanping Tian, Hongli Li

**Affiliations:** 1 Department of Histology and Embryology, Third Military Medical University, Chongqing 400038, China; E-Mails: matt0119@163.com (T.M.); wuxiyan2003@163.com (X.W.); fengcai1112@126.com (Q.C.); yunwang30@hotmail.com (Y.W.); xiaolan35@hotmail.com (L.X.); 2 Battalion 7 of Cadet Brigade, Third Military Medical University, Chongqing 400038, China

**Keywords:** Pb exposure, oligodendrocytes, differentiation, sodium/calcium exchanger 3, calcium signal

## Abstract

Lead (Pb) poisoning has always been a serious health concern, as it permanently damages the central nervous system. Chronic Pb accumulation in the human body disturbs oligodendrocytes (OLs) differentiation, resulting in dysmyelination, but the molecular mechanism remains unknown. In this study, Pb at 1 μM inhibits OLs precursor cells (OPCs) differentiation via decreasing the expression of Olig 2, CNPase proteins *in vitro*. Moreover, Pb treatment inhibits the *sodium/calcium exchanger 3* (*NCX3*) mRNA expression, one of the major means of calcium (Ca^2+^) extrusion at the plasma membrane during OPCs differentiation. Also addition of KB-R7943, *NCX3* inhibitor, to simulate Pb toxicity, resulted in decreased myelin basic protein (MBP) expression and cell branching. Ca^2+^ response trace with Pb and KB-R7943 treatment did not drop down in the same recovery time as the control, which elevated intracellular Ca^2+^ concentration reducing MBP expression. In contrast, over-expression of *NCX3* in Pb exposed OPCs displayed significant increase MBP fluorescence signal in positive regions and CNPase expression, which recovered OPCs differentiation to counterbalance Pb toxicity. In conclusion, Pb exposure disturbs OLs differentiation via affecting the function of *NCX3* by inducing intracellular calcium overload.

## 1. Introduction

Lead (Pb) poisoning has been a serious public health problem worldwide, as it causes permanent nervous system damage [1]. Excessive accumulation of Pb in human bodies leads to catastrophic conditions such as infertility [2], behavioral changes [3], *etc.* Its devastating effect on central nervous system (CNS) is the most important problem [4]. Acute Pb exposure of high dose is associated with neural cell apoptosis in hippocampus *in vivo* [5]. However, chronic low doses of Pb exposure result in behavioral and cognition alteration [6]. The molecular mechanism of Pb poisoning symptoms in CNS is due to white matter impairment [7] and serious disturbance of myelin sheath formation [8]. Among myelination-related glia cells, oligodendrocytes (OLs) are more sensitive and vulnerable to Pb [9]. Pb directly delays the differentiation of oligodendrocytes progenitor cells (OPCs) in cultured OLs. However, the molecular mechanism of Pb toxicity remains unknown.

During CNS development, OPCs need to pass through four stages to become mature myelin-forming cells. Abnormalities or injury in OLs usually leads to demyelination disease [10,11,12]. Additionally, several studies have addressed the importance of calcium (Ca^2+^) signaling in OLs differentiation and myelination. Changes in intracellular Ca^2+^ levels not only influence the transition of OPCs into mature myelinating OLs, but also intervene in the initiation of myelination and remyelination processes [13]. Thus, among Pb-related damages, the abnormal elevation of Ca^2+^ levels is crucial. As maintaining Ca^2+^ homeostasis is critical for the viability and function of OLs, disturbance of Ca^2+^ homeostasis is a hallmark of damage. However, reports assessing the mechanism of Pb induced disturbance of Ca^2+^ are scarce.

Recent studies have shown that Pb enhances the generation of reactive oxygen species and reduces the antioxidant defense system of cells [14,15], thus resulting in the decreased expression of Ca^2+^ extrusion proteins. As a result, accumulation of oxidative stress occurs in the cell which subsequently interferes with intracellular Ca^2+^ homeostasis causing cellular damage. One of the major means of Ca^2+^ extrusion at the plasma membrane of many excitable and non-excitable cells is the *sodium*/*calcium exchanger* (*NCX*), which helps in rapid recovery from high intracellular Ca^2+^ concentrations and protects cells from Ca^2+^ overload and eventual death. Although the expression of the different *NCX* mRNAs in OLs has been investigated [16], their role in Pb-induced Ca^2+^ elevation followed by OLs damage has not yet been investigated.

Three different *NCX* genes (*NCX1*, *NCX2* and *NCX3*) have been reported in mammals [17,18]. The importance of *NCX3* in the regulation of the physiological and pathological functions of the CNS has been widely recognized [19]. Boscia *et al.* reported that silencing or knocking out of *NCX3*, but not *NCX2* and *NCX1*, impairs OLs differentiation [20]. As dysregulation of Ca^2+^ homeostatic mechanisms is an important feature of Pb toxicity and *NCX3* contributes to Ca^2+^ influx. The present study sought to determine the extent to which inhibition of *NCX3* may affect Pb toxicity in OLs lineage. We assessed the functional activity of *NCX3* during OLs development in primary OPCs cultures. The results demonstrated that expression of *NCX3* is strongly down-regulated in the Pb-exposed OLs, which impairs OLs differentiation, resulting in dysmyelination. Furthermore, the over-expression of *NCX3* reversed Pb-induced disturbances of oligodendrocytes differentiation. These findings provide an important insight into the molecular mechanism of Pb toxicity on OLs.

## 2. Results

### 2.1. Discrepancies in Differentiation of OLs Precursor Cells (OPCs) at Low Concentrations of Pb in Vitro

**Figure 1:**
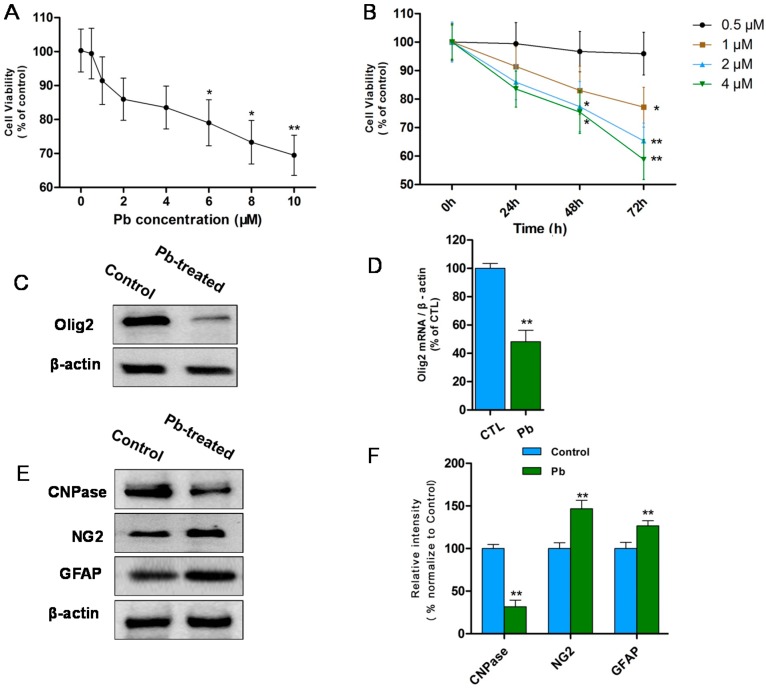
(**A**) MTT assay (0.5–10 μM Pb acetate for 24 h) showed that cell viability decreases in a dose-dependent manner from 1 to 10 μM. Significant differences appeared after concentration reached 6 μM. The values represents the mean ± S.E.M. (standard error of the mean) (*n* = 3). * *p* < 0.05; ** *p* < 0.01 *vs.* control group; (**B**) MTT assay (0.5–4 μM Pb acetate for 0–72 h) revealed that 1 μM Pb notably decreases cell viability at 72 h and 2, 4 μM Pb caused significant reduction at 48 h. The values represent the mean ± S.E.M. (*n* = 3). * *p* < 0.05; ** *p* < 0.01 *vs.* control group; (**C**) Western blot analysis revealed that Olig2 protein levels decreased after treated with 1 μM Pb (24 h) and differentiated for 3 days compared with controls; (**D**) Relative quantification of Western blot analysis is depicted in the bar graphs. The values represent the mean ± S.E.M. (*n* = 3). ** *p* < 0.01 *vs.* Control group; (**E**) Western blot analysis showed reduction in CNPase protein and NG2, GFAP increased after treated with 1 μM Pb (24 h) and differentiated for 3 days compared with controls; and (**F**) Relative quantification of Western blot analysis is depicted in the bar graphs. The values represent the mean ± S.E.M. (*n* = 3). ** *p* < 0.01 *vs.* control group.

To explore Pb toxicity on OPCs *in vitro*, MTT (3-[4,5-dimethylthiazol-2-yl]-2,5 diphenyl tetrazolium bromide) assay was performed to determine the cell viability (Figure 1A,B). OPCs were exposed to Pb at different concentration (0–10 μM) for 24 h (Figure 1A). Results showed that when Pb concentration increased from 1 to 10 μM, cell viability of OPCs began to decrease in a dose-dependent manner and a significant decrease was recorded after concentration reached 6 μM (*p* < 0.05; Figure 1A). It indicates that Pb at high concentration (≥6 μM) is cytotoxic to OPCs and decreases cell viability *in vitro*.

In order to amplify Pb-induced injury on OPCs at low concentrations (0.5, 1, 2, 4 μM), the reaction time of MTT experiments were prolonged from 24 to 72 h (Figure 1B). It has been observed that 0.5 μM Pb did not affect cell viability even at 72 h. However, 1 μM Pb significantly decreased cell viability at 72 h and significant decreases were also observed with 2 or 4 μM Pb for 48 h (*p* < 0.05; Figure 1B). This indicates that Pb exposure, even at low concentration, also induces potential injuries in OPCs, which get accumulated in long term exposure, leading to cell death. 1 μM Pb was a typical concentration to explore the effects of Pb [21], which did not kill cells, but caused potential injuries.

**Figure 2:**
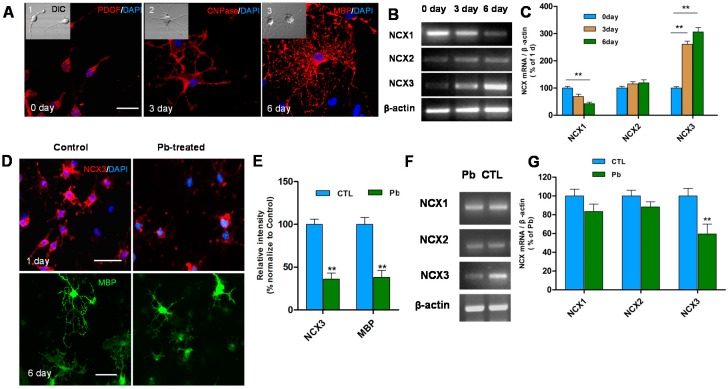
(**A**) Differentiated OLs were identified by immunofluorescence staining. PDGFα (an OPCs marker), CNPase (a developing OLs marker) and MBP (a mature OLs marker) demonstrates the morphologic characteristics of OPCs exposed to differentiation medium 0 day (**A1**), 3 day (**A2**) and 6 day (**A3**) with Differential Interference Contrast (DIC) pictures on the top left. Scale bar = 50 μm; (**B**) RT-PCR shows the expression levels of three types of *NCX* mRNA at 0, 3 and 6 day after exposure to differentiation medium; (**C**) Relative quantification of RT-PCR analysis is depicted in the bar graphs. The values represent the mean ± S.E.M. (*n* = 3). ** *p* < 0.01 *vs.* control group; (**D**) *NCX3* (red) positive cells decreased in OPCs exposed to differentiation medium one day after Pb treatment. Scale bar = 100 μm. MBP (green) positive cells were smaller with fewer processes compared to untreated controls. Scale bar = 50 μm; (**E**) Relative quantification of *NCX3* and MBP staining is depicted in the bar graphs. The values represents the mean ± S.E.M. (*n* = 5). ** *p* < 0.01 *vs.* control group; (**F**) RT-PCR showed the significant reduction in expression of *NCX3* mRNA after 3 day with Pb exposure (24 h) and no obvious changes were observed in *NCX1*, *NCX2*; and (**G**) Relative quantification of RT-PCR analysis is depicted in the bar graphs. The values represents the mean ± S.E.M. (*n* = 3). ** *p* < 0.01 *vs.* control group.

To further explore Pb-induced injury in OLs lineage, the differentiation of OPCs were observed with and without 1 μM Pb treatment. Firstly, the transcription factor Olig2 which plays a crucial role in OLs differentiation and maturation was examined by Western blot *in vitro* (Figure 2C). The expression of Olig2 protein levels decreased in cultures developing OLs treated with 1 μM Pb compared with controls (*p* < 0.01; Figure 1C,D). Then, the expression of differentiation associated factors was detected by Western blot. After 1 μM Pb treatment, the expression of CNPase were significantly decreased in OLs (*p* < 0.05; Figure 1E,F). However, the expression of NG2 and GFAP were increased apparently compared with the control (*p* < 0.01; Figure 1E,F). It indicates that OPCs was mostly delayed at the immature stage in medium after Pb treatment. Additionally, in our culture system, part of OPCs differentiated into astrocyte and they were activated by Pb, resulting in elevated GFAP expression. In short, results suggest that Pb at low concentration (1 μM) disturbs the differentiation of OPCs *in vitro*.

### 2.2. Pb Exposure Decreases the Expression of NCX3 and MBP in OLs Differentiation Model

To understand the mechanism of Pb-induced dysmyelination and the function of *NCXs* during differentiation of OPCs, the expression of *NCXs* was detected after being cultured in normal differentiation medium with and without 1 μM Pb treatment. OPCs were differentiated in differentiation medium for 1, 3 and 6 day. Expression of PDGFα (a marker of OPCs), CNPase (a developing OLs marker), and MBP (a mature OLs marker) were assessed to identify the differentiation stage of OLs by immunofluorescence staining (Figure 2A). In normal differentiation medium, OLs were differentiated into OPCs, developed into OLs and matured respectively at 1, 3 and 6 day. Then, RT-PCR was performed to reveal the expression of *NCXs* during differentiation of OPCs (Figure 2B). A significant increase in *NCX3* mRNA transcripts at three and six days in normal OLs differentiation medium (*p* < 0.05; Figure 2B,C) was observed. However, *NCX1* expression progressively decreased six days after exposure to differentiation medium (*p* < 0.05; Figure 2B,C). No changes in *NCX2* transcripts were observed during OLs development (*p* > 0.05). This suggests that *NCX3* plays a crucial role in OLs maturation in normal differentiation medium, but not *NCX1* or *NCX2*.

Further, to detect whether Pb toxicity affects the function of *NCXs*, the transcripts were observed and significant reduction in *NCX3* mRNA was reported (*p* < 0.05; Figure 2F,G). However, there was no influence on the expression of *NCX1* and *NCX2*. The down-regulation of *NCX3* expression suggests that the function of *NCX3* is inhibited during Pb exposure.

The influence of *NCX3* inhibition on OLs generation was examined and has been found that *NCX3* immunoreactivity intensely decreased in OPCs treated with Pb and differentiated for one day (*p* < 0.01; Figure 2D,E). The morphologically-differentiated OLs that were treated with Pb and differentiated for six days had fewer primary processes and less arborization than untreated cells (Figure 2D). These differentiated OLs exhibited MBP immunofluorescence signal which dropped down clearly (*p* < 0.01; Figure 2D,E). Therefore, our results suggest that the morphological maturation of Pb-treated differentiated OLs was inhibited, accompanied with reduction in *NCX3* expression, thus suggesting an important role of *NCX3* in regulation of OLs development.

**Figure 3:**
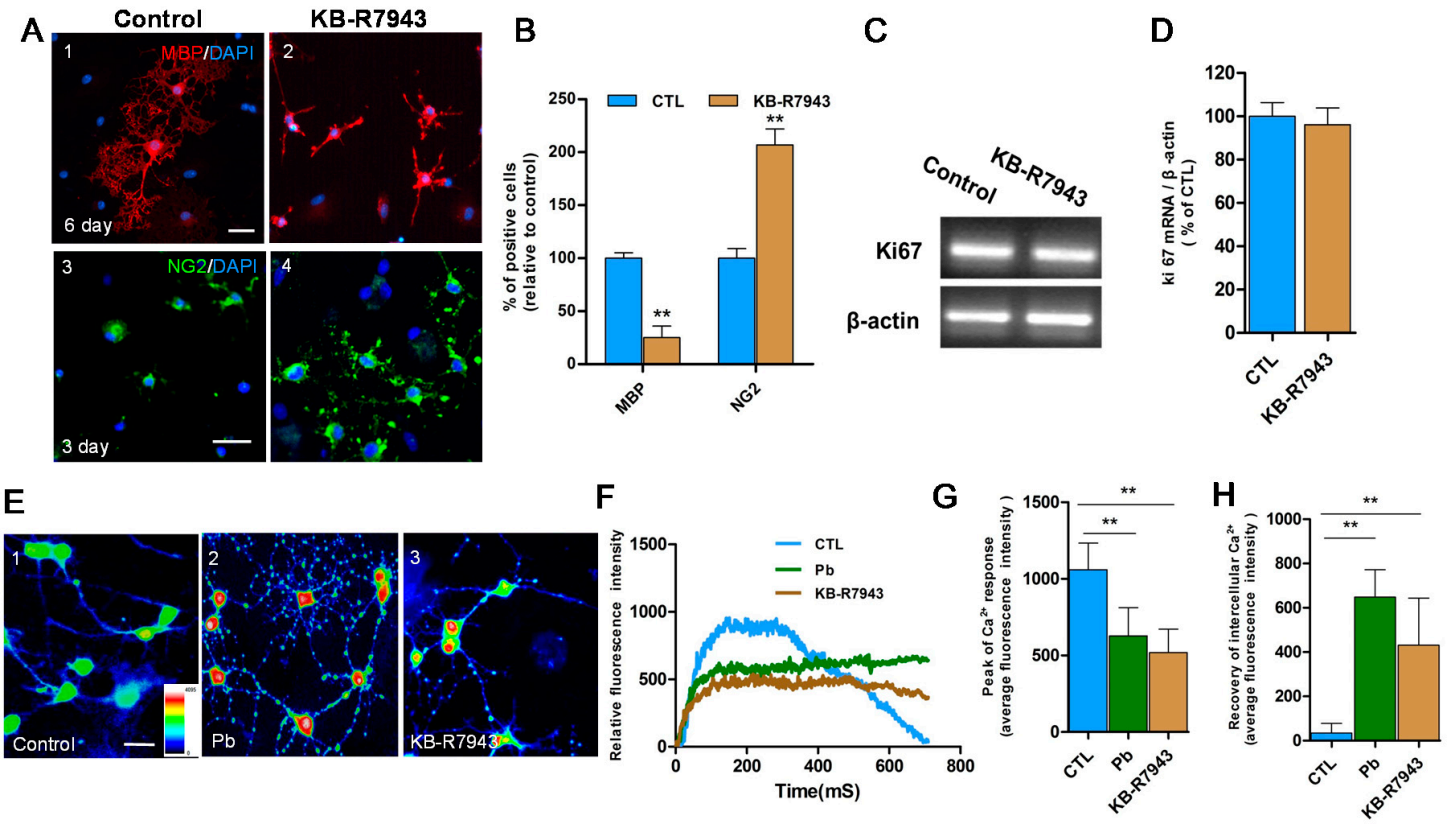
(**A**) Confocal immunofluorescence images showed MBP (red) and branches of cells were decreased but NG2 immunosignal (green) was increased in OPCs exposed to differentiation medium with KB-R7943 (*NCX3* inhibitor, **A2**,**A4**) compared with that of controls (**A1**,**A3**); (**B**) Relative quantification of the staining of MBP and NG2 is depicted in the bar graphs. The values represents the mean ± S.E.M. (*n* = 5). ** *p* < 0.01 *vs.* control group; (**C**) RT-PCR results showed mRNA expression of antigen Ki67 was not changed in OPCs in differentiation medium with and without KB-R7943; (**D**) Relative quantification of the mRNA expression of antigen Ki67 is depicted in the bar graphs. The values represents the mean ± S.E.M. (*n* = 3); (**E**) Intracellular Ca^2+^ levels at the resting condition represented with pseudo-color signals were measured by Fluo-3 video imaging after 3 day exposure to differentiation medium. Pseudo-color signals were only observed in the somatic region in normal controls (green and blue; **E1**). With Pb (**E2**) and KB-R7943 (**E3**) treatments, increased signals (red and yellow) were observed in the soma and processes; (**F**) Following 20 mM K^+^ stimulation, representative Ca^2+^ response traces were recorded in cells treated with Pb (green trace) or KB-R7943 (brown trace) and control cells (blue trace); (**G**,**H**) Relative quantification of peak of Ca^2+^ response and recovery of intracellular Ca^2+^ is depicted in the bar graphs. The values represents the mean ± S.E.M. *n* = 30 from three independent experiments. ** *p* < 0.01 *vs.* control group. Scale bar = 50 μm.

### 2.3. Pb Induced Inhibition of NCX3 and Increased Intracellular Calcium Interfering with OPCs Differentiation

To examine whether Pb-induced inhibition of *NCX3* activity could influence OLs generation, *NCX3* protein activity was blocked using 100 μM KB-R7943, to simulate the effects of Pb and then assessed the differentiation in OPCs (Figure 3). The results showed decreased MBP expression and cell branching in cells after six days in differentiation medium after KB-R7943 treatment (*p* < 0.01; Figure 3A,B), however, the number of NG2^+^ cells was significantly higher than the controls (*p* < 0.01; Figure 3A,B). Proliferation of OPCs was also detected by Ki67 expression but no notable difference was found in expression levels between cells treated with KB-R7943 and controls (*p* > 0.05; Figure 3C,D).

To test the presence of functional *NCX3*, intracellular Ca^2+^ fluorescent signals and activity responses were assessed in differentiated OLs (3 days of differentiation medium) by Fluo-3 video-imaging. Firstly, intracellular Ca^2+^ concentrations at resting condition were recorded and distinguished using pseudo-color signals. Pseudo-color signals were observed weakly in the somatic region in normal controls (Figure 3E1), but increased pseudo-color signals in perinuclear areas of OLs after Pb were observed (Figure 3E2) and after KB-R7943 (*NCX3* inhibitor; Figure 3E3) treatments. Many punctuated signals were found in processes of OLs (Figure 3E2,E3) in the two groups. Pb or KB-R7943 treatments resulted in an elevation of basal intracellular calcium concentrations (Figure 3E).

Ca^2+^ response traces were also recorded followed by 20 mM K^+^ stimulation. The Ca^2+^ response curve increased quickly, and then dropped back to baseline levels in normal OLs (Figure 3F blue). However, in OLs treated with Pb or KB-R7943 (*n* = 30 from three independent experiments), the trail of the elevated curve did not drop back down to baseline levels (Figure 3F green and brown) in the same recovery time. The average fluorescent intensity of the Ca^2+^ recovery stage remained significantly stronger compared with normal controls (*p* < 0.01; Figure 3H). However, the peak of the Ca^2+^ response was smaller compared to controls (*p* < 0.01; Figure 3G). Treatment with Pb or KB-R7943 not only decreased the amplitude of Ca^2+^ response, but also prevented the recovery of calcium concentrations by K^+^ stimulation. The results demonstrated that the relative intracellular Ca^2+^ intrusion and extrusion was inhibited by Pb treatment, especially extrusion function dysregulation.

### 2.4. Overexpression of NCX3 Reverse Pb Induced Disturbances of OLs Differentiation

**Figure 4:**
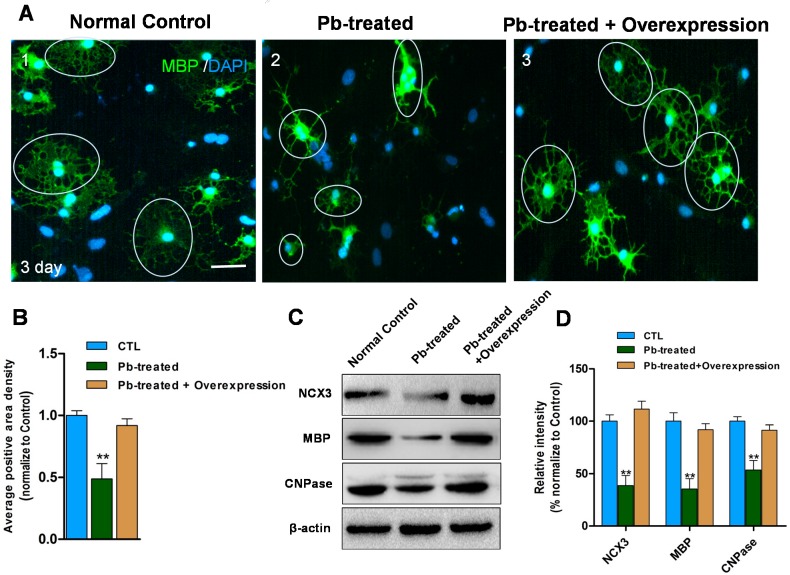
(**A1**) MBP immunosignal (green) in 3 day differentiated oligodendrocytes transfected with empty control vector (cells in circle). (**A2**) Decreased MBP immunosignal (green) in 3 day differentiated oligodendrocytes after 1 μM Pb for 24 h transfected with empty control vector (cells in circle). (**A3**) Recovered MBP positive membrane area in *NCX3* overexpression Pb-treated cells (cells in circle) compared with (**A2**); (**B**) Relative quantification of average area density in each cell is depicted. Values are obtained from three independent experiments with 10 cells recorded for each experiment. ** *p* < 0.01 *vs.* control group; (**C**) Western blot analysis shows *NCX3*-overexpressing increased *NCX3*, MBP and CNPase protein levels in Pb-treated OLs compared with cells transfected with empty vector; (**D**) Relative quantification of Western blot analysis is depicted in the bar graphs. The values represents the mean ± S.E.M. (*n* = 3). ** *p* < 0.01 *vs.* control group, Scale bar = 50 μm.

To investigate whether the expression of *NCX3* was sufficient to induce the expression of myelin proteins in Pb-exposed OLs, OPCs were transfected with either a plasmid encoding *NCX3* or an empty control vector to detect MBP expression and positive regions, following 1 μM Pb treatment (Figure 4). The results showed after three days of differentiation, average intensity of MBP positive regions (*n* = 30 from three independent experiments) were significantly decreased in Pb-treated OLs (*p* < 0.01; Figure 4A,B). However, Pb-treated cells transfected with *NCX3* had significantly up-regulated average intensity of MBP positive regions compared with those transfected with an empty vector. Additionally, *NCX3* over-expression in Pb-treated cells led to recovered expression of MBP proteins, similar with the control group. Western blot analysis also confirmed that over-expression of *NCX3* increases the expression of MBP and CNPase in Pb-exposed OLs (*p* < 0.01; Figure 4C,D).

## 3. Discussion

Pb is an immobile toxic metal that accumulates in the human body, leading to chronic poisoning through the food chain [22]. Children are most susceptible group [23]. Thirty-two studies published during 1994–2004 showed that mean blood lead level (BLL) of Chinese children was 9.3 μg/L, with 33.8% of them had BLLs above 10 μg/L [24], which is the threshold for defining lead poisoning in children [25]. Pb pollution is an urgent and serious problem to be looked after throughout the world [26]. Chronic lead poisoning is also associated with fatal encephalopathy. As Pb can pass through the blood-brain barrier and a greater proportion of systemically-circulating Pb gains access to the brain of children, especially those of five-year age or younger, thus making the developing brain vulnerable to Pb toxicity [8]. The CNS symptoms of Pb toxicity include behavioral and cognition alteration due to white matter impairment [7]. In addition, Pb induces neural cell apoptosis and influences neurotransmitter storage and release processes. Studies also revealed that Pb has a toxic effect on oligodendorocyte lineage. Deng *et al.* directly proved that Pb delays the differentiation of OPCs *in vitro* [21] and Coria *et al.* proved that Pb causes hypomyelination and demyelination *in vivo* [27]. Our results of MTT assay also provides evidence for Pb toxicity, as Pb of high concentration (≥6 μM) significantly decreases OPCs viability and Pb of relatively low concentration disturbs OPCs differentiation via decreasing CNPase expression and increased NG2 and GFAP expression. This indicates OPCs were mostly delayed at an immature stage after Pb treatment. Additionally, as part of OPCs differentiated into astrocytes, Pb-induced injury activated astrocytes in the culture system and led to elevation of GFAP expression. In short, Pb treatment disturbed the differentiation of OPCs and expression of differentiation associated factors.

OLs are the myelinating glial cells of the CNS. OPCs differentiate into myelin-forming OLs via cascade of distinct developmental steps: the formation of the OPCs, their differentiation to pre-myelinating OLs and, finally, the development of mature myelinating OLs. The marker identifying the different stages of OLs was assessed in the current study. Results suggested MBP (marker of mature OLs) was significantly decreased in cultured OPCs with 1 μM Pb and OLs presented immature features compared with the control group. Furthermore, to explore the molecular mechanism of Pb-induced damage on OLs, we investigated expression of *NCX*, which were potentially affected by Pb-induced oxidative stress. Our results also provided evidence for the hypothesis that *NCX3* notably decreased in Pb-exposed OLs, underlying the importance of *NCX3* function in the differentiation of OPCs.

*NCX* is a bidirectional membrane ion transporter that couples the influx/efflux of Ca^2+^ to the efflux/influx of Na^+^ and regulates the levels of intracellular Ca^2+^ [28]. Three different *NCX* genes (*NCX1*, *NCX2*, and *NCX3*) have been identified in mammals [17,18]. The importance of *NCX3* in the regulation of physiological and pathological functions of neural cells has been widely recognized. Evidence indicates that *NCX3* silencing by RNA interference increases vulnerability of cerebellar granule neurons to Ca^2+^ overload [29]. Our results demonstrated that pharmacologically-inhibited *NCX3* significantly decreased the expression of MBP, however, increased the expression of NG2^+^ (marker for OPCs) in cultured OLs. Inhibition of *NCX3* directly affects the differentiation of OPCs causing development arrest at immature stage. In addition, no alteration was observed in the level of Ki67, indicating that Pb toxicity did not influence the survivor of OPCs, but delayed its differentiation. Our study has also reported the involvement of downstream Ca^2+^ signaling mediated by *NCX*3 during OLs development. Inhibition of *NCX3* notably increased intracellular Ca^2+^. Thus, the changes in intracellular Ca^2+^ levels regulated by *NCX3* influences the developmental processes of OPCs into mature OLs [20]. When *NCX3* was overexpressed in Pb-exposed OLs, the expression of MBP and CNPase proteins were partly recovered, indicating the crucial role of *NCX3* in Pb-induced injury. It also provided evidence for therapy and prevention of Pb poisoning as well as instructions for drug development.

Numerous studies have found that a lethal influx of Ca^2+^ can occur in many cell types as a consequence of receptor overstimulation and exposure to cytotoxic agents [30]. OLs, like neurons, are vulnerable to Ca^2+^ overload resulting from dysregulation of channels or pumps, resulting in either apoptotic or necrotic cell death. Utilizing live cell imaging, we also observed that intracellular Ca^2+^ levels increased persistently (resulting in Ca^2+^ overload), without returning to baseline levels after treatment with Pb or the *NCX3* blocker. Changes in Ca^2+^ channel expression during OLs development are functionally linked to the different developmental stages of myelination. The expression of *NCX3* in Pb-exposed OLs decreased, leading to a change in Ca^2+^ homeostasis. These changes can then cause Ca^2+^ overload, which will cause OLs injury.

Due to the special properties of flexible coordination chemistry, high affinity for carboxylate oxygen, and effects on fluidity and fusion of cellular membranes, Ca^2+^ ions eagerly interact with various biological molecules. However, high Ca^2+^ concentrations, at the same time, are harmful for biological materials, as excess Ca^2+^ causes the aggregation of nucleic acids and affects the expression of genes, especially transcription factors [31]. The formation of OLs from their precursors requires activation and coordination of a set of stage-specific transcriptional regulators that are important for the biosynthesis of myelin components [32]. The basic helix-loop-helix (bHLH) transcription factor, Olig2, plays a crucial role in OLs differentiation and myelination, as well as remyelination [33,34,35,36]. Lacking Olig2 expression inhibits NG2^+^ cell development at embryonic and perinatal stages, which can be rescued by the addition of a transgene containing the Olig2 locus [36]. Olig2 expressing OPCs preferentially differentiate into OLs during remyelination in cuprizone induced demyelinated models [37,38]. In the present study, Pb treatment also decreased Olig2 expression in cultured cells. Together, our data *in vitro* provides further evidence that elevation of intracellular concentrations of Ca^2+^ resulting from down-regulation of *NCX3* expression by Pb treatment inhibits expression of Olig2, reducing OPCs differentiation.

Overall, the results of the present study suggest that in Pb-exposed OPCs, the down-regulation of *NCX3* results in intracellular Ca^2+^ overload which, in turn, affects the expression of other genes involved in OPCs differentiation, such as the transcription factor Olig2. In contrast, overexpression of *NCX3* in Pb-exposed OPCs counterbalanced injuries. Therefore, the results in the present study suggest that a critical step in the molecular mechanism of Pb toxicity is on OLs.

## 4. Experimental Section

### 4.1. OPCs Culture and Differentiation

The rat OPCs were propagated [39]. At first, mixed cells were isolated from the cortex region of postnatal day 1–3 (P1–P3) SD rats and cultured in OPC-proliferation medium (DMEM/F12 + 15% B104-conditioned medium+ 1% N2-supplement (Invitrogen, Carlsbad, CA, USA) for 5–7 days. The cells were then incubated with OPC-isolation medium (DMEM/F12 + 0.01% EDTA + 5 mg/mL insulin) at room temperature (25 °C) for 15–30 min and centrifuged (1000 r/min) for 5 min. The pellets were suspended with the OPC-proliferation medium and seeded into dishes or cover slips coated with poly-d-lysine for experiments. The OPCs were induced to differentiate by OPC differentiation medium (DMEM/F12 + 1% N2-supplement + 5 mg/mL *N*-acetyl-l-cysteine (Amresco, Solon, OH, USA) + 1% fetal bovine serum + 5 mg/mL insulin). At the indicated time points, the cells were used for respective tests.

### 4.2. MTT Assay

The rat OPCs cell line (1 × 10^4^/well) were seeded in 96-well plates and cultured for 6 h at 37 °C. Pb acetate was added in increasing concentrations (0.5–10 μm) and incubated for 24–72 h, followed by addition of 20 μL MTT (5 mg/mL in PBS; Beyotime, Nantong, China) for another 4 h. then the supernatant was discarded and 150 μL DMSO was used to dissolve formazan. The OD for each well was measured at 540 nm using a SpectraMax M2e spectrophotometer (Molecular Devices, Sunnyvale, CA, USA).

### 4.3. Confocal Ca^2+^ Imaging

The OPCs/OLs were grown and differentiated in glass-bottom dishes treated and untreated with KB-R7943 (100 μM, blocker of *NCX3*) or 1 μm Pb acetate for three days, then incubated in imaging wash buffer (135 mM NaCl, 2 mM glucose, 8 mM HEPES, 2 mM MgCl_2_, 3 mM KCl, and 2.2 mM CaCl_2_; pH 7.4) containing 5 μM fluo-3 AM for 20 min at 37 °C. After incubation, the cells were maintained for at least 20 min at room temperature (RT) in fresh imaging wash buffer for complete dye de-esterification followed by qualitative evaluation of intracellular Ca^2+^ level in cells at the resting condition or the changes in its activity by 20 mM K^+^ stimulation under confocal laser scanning microscope. Fluorescence images were acquired and measured through confocal laser scanning microscope (Olympus, IX81, Tokyo, Japan) and Fluoview image processing software (v2.1, Olympus). The experiment was carried out in triplets with 10 cells recorded for each experiment.

### 4.4. Immunohistochemistry and Immunofluorescent Staining

For immunohistochemical staining, the dishes were treated with 0.3% hydrogen peroxide in methanol for 15 min, and then incubated with a blocking solution composed of 5% bovine serum albumin in PBS at room temperature for 1 h. Then, cells were incubated in primary antibodies overnight at 4 °C followed by the fluorescence-conjugated secondary antibodies incubated at room temperature for 1 h. Cell nuclei were stained with DAPI (0.1 μg/mL in PBS). The immunoreactivity was determined using confocal laser-scanning microscope (Olympus, IX81). Morphological features of single cells were recorded using differential interference contrast (DIC) under confocal microscopy.

### 4.5. RT-PCR

Total RNA of cultured cells was collected using the Trizol reagent (Life Technology, Carlsbad, CA, USA) according to the manufacture’s manual. Single strand cDNA was synthesized from the total RNA using random primer and SuperScriptase III (Life Technology). The specific primers of target genes were as follows: *NCX1*: 5ʹ-CTGGAGCGCGAGGAAATGTTA-3ʹ and 5ʹ-GACGGGGTTCTCCAATCT-3ʹ; *NCX2*: 5ʹ-AGGAGGCCGCACACCTTTCC-3ʹ and 5ʹ-CAAGGCGTGGCTGGGCTCTC-3ʹ; *NCX3*: 5ʹ-GGCTGCACCATTGGTCTCA-3ʹ and 5ʹ-GACGGGGTTCTCCAATCT-3ʹ; Ki67: 5ʹ-AAGAGTGAGGGAATGCCTAT-3ʹ and 5ʹ-GCTTTCTTGGGAATGTCTGT-3ʹ; *β-actin*: 5ʹ-CGTTGACATCCGTAAAGACC-3ʹ and 5ʹ-CATCGTACTCCTGCTTGCT-3ʹ. The optical density of the bands of PCR products (normalized with those of *β-actin*) was determined by Image Pro Plus image analysis system (Version 5.1, Media Cybernetics, Silver Spring, MD, USA).

### 4.6. Western Blot Analysis

Protein samples were extracted from cultured cells using RIPA (Radio Immunoprecipitation Assay) lysis buffer with freshly 1% PMSF solution (Biocolors, Shanghai, China). These proteins were then denatured and separated using SDS-PAGE, followed by electrophoresis; the proteins were transferred to polyvinyldifluoride membranes. The membranes were incubated overnight at 4 °C with the primary antibodies and then incubated with HRP-linked secondary antibodies. Immunoreactive bands were detected using an ECL plus detection kit (ECLplus, GE Healthcare, Little Chalfont, UK). The optical density of the bands (normalized with those of β-actin) was determined by Image Pro Plus image analysis system and the protein determination was done using a Coomassie Brilliant Blue G250 (Boster, Beijing, China).

### 4.7. OPCs Transfection

Rat *NCX3* gene was subcloned into pCMV-N-His vector. The control plasmid pCMV-N-His and pCMV-*NCX3* were transfected into OPCs (4.5 × 10^4^ cells per well) using Lipofectamine 3000 (Invitrogen) for 24 h and the cells were cultured in OPC-Differentiation Medium for three days.

### 4.8. Quantitive Image Analysis

For Statistical analysis, at least nine representative fields were randomly acquired at 20× magnification from each of the two experiments performed in triplicate. Cell counting was conducted on nine randomly-chosen fields for each sample. Quantification of immunostaining and cell counting was performed using the Image Pro Plus software. The optical density of the bands of PCR products or Western blot results (normalized with those of *β-actin*) was also determined by Image Pro Plus image analysis system.

### 4.9. Statistical Analysis

Statistical analyses were performed using one- or two-way analysis of variance (ANOVA) followed by Tukey’s *post hoc* test. Comparisons between two experimental groups were made using Student’s *t*-test. A significant statistical difference was determined by a value of at least *p* < 0.05.
